# Supplementary material for: Logarithmic distributions prove that intrinsic learning is Hebbian
Source: F1000Res. 2017 Oct 11;6:1222. Originally published 2017 Jul 25. [Version 2] doi: 10.12688/f1000research.12130.2 (PMC5639933; doi:10.12688/f1000research.12130.2)
Supplement: Peer review history

# Peer review: Referee response for version 2

**DOI:** 10.5256/f1000research.13939.r26899

**Published:** 2017-10-12

**Authors:** Rune W Berg

**Affiliations:** 1 Department of Neuroscience, University of Copenhagen, Copenhagen, Denmark

The author has appropriately improved the manuscript. I have no further comments.

I have read this submission. I believe that I have an appropriate level of expertise to confirm that it is of an acceptable scientific standard.

# Peer review: Referee response for version 1

**DOI:** 10.5256/f1000research.13128.r25241

**Published:** 2017-09-25

**Authors:** Tomáš Hromádka

**Affiliations:** 1 Institute of Neuroimmunology, Slovak Academy of Sciences, Bratislava, Slovakia

In the manuscript the author documents various instances of lognormal distributions of several neuronal parameters across brain areas. The description then serves as motivation for an intriguing investigation of possible underlying mechanisms responsible for creating such distributions. In particular, combination of Hebbian learning with homeostatic adaptation in a simple model is sufficient to recreate the observed distributions of firing rates, synaptic weights, and gains.

 The presented combination of data and modeling is interesting. The model itself is presented in sufficient detail. However, some statements throughout the text seem to be too strong given the evidence. I suggest rephrasing the strong statements and conclusions to better reflect the presented data and model. After minor changes the manuscript certainly will be of interest to experimenters as well as modelers.

**Comments:**

*1. Strong statements*

 Many a strong statement is sprinkled throughout the manuscript. I suggest modifying such statements to reflect evidence presented in the manuscript itself and the cited literature.

 For example:

 Abstract: "Logarithmic scale distribution of weights and gains appears as a functional property that is present everywhere." The author probably meant to say "everywhere we looked."

 Introduction: "...lognormal distributions can only be maintained by Hebbian-style, not homeostatic adaptation." There might be a plethora of other mechanisms (not studied in the manuscript) that could lead to lognormal distributions. The fact that Hebbian learning is sufficient does not mean it is also necessary.

 Similarly, here: "The answer is that the attested distribution of intrinsic gain can only derive from a Hebbian style adjustment rule, even though additional homeostatic adaptation is possible. Intrinsic plasticity is Hebbian".

*2. Methods section*

 The Methods section presents a mix of Methods, Introduction, and Results. I suggest refining the section and moving some results into Results. For example, descriptions of various datasets could be easily incorporated into Results, especially when they actually contain new data - "We extend this dataset by recordings from different types of cortical

 neurons..." (p. 3).

*3. Lognormal fits*

 Presented distributions of various neuronal parameters do appear lognormal, with a few exceptions, as correctly pointed out in the text. It is also unlikely that exponential distribution would provide a better fit to any of presented examples. There are, however, other distributions which *might*, or might not, fit the data better? Was the goodness-of-fit evaluated in any way, or were other types of distributions considered? A brief statement along these lines would further improve this interesting manuscript.

*4. Minor comments*

 p. 3 - "The distribution of spike rates for neurons spiking in the absence of synaptic input...". I believe it is highly unlikely that spontaneous rates in-vivo arise in the absence of synaptic inputs. Unless controlled---such as in slices, in neuronal cultures, etc.---neurons will receive barrages of input activity even in the absence of experimental stimulation.

 p. 7 - "...populations I, J with each n = 1000 neurons..." should be "...populations I, J each with n = 1000 neurons..."

 p. 8 - "The output R^J may BE used as input..."

*5. Minor changes to figures*

 Fig. 9 caption needs description of color arrows. A simple statement, such as "J (excitatory, blue arrows) and H (inhibitory, red arrows)", would suffice.

 What is the meaning of colors in Figs. 10 and 11? If there is any, please state so in the caption. If there isn't, please state so as well. The two 'sheets' in Fig. 10 are almost indistinguishable. If colors have no results-related meaning, it might be helpful to plot the bottom sheet (for example) in slightly different colors.

I have read this submission. I believe that I have an appropriate level of expertise to confirm that it is of an acceptable scientific standard.

# Peer review: Referee response for version 1

**DOI:** 10.5256/f1000research.13128.r25048

**Published:** 2017-08-29

**Authors:** Rune W Berg

**Affiliations:** 1 Department of Neuroscience, University of Copenhagen, Copenhagen, Denmark

In this study the author investigate various distributions of neuronal networks, and suggest that lognormality is ubiquitous. The author constructs a Hebbian model to better understand the mechanisms behind these distributions. The analyses are detailed, well-done and interesting. The manuscript is not so well--organized and the writing can be improved. There are many statements, which are stronger than the data can support. I suggest a thorough rewrite - incorporating the issues below.

**Major concerns:**

 Methods section is full of results, discussions and conclusions. I suggest a thorough rewrite of the manuscript to better separate out the methods, results and discussion.

**From the discussion:**

 “Again the observation of lognormal distributions is ubiquitous. We find synaptic weight distributions for cortex ^6^ and cerebellum that are lognormal, with characteristic width of distributions. For intrinsic properties, striatal projection neurons and cortical neurons ^23^ show responses to constant current and current- to-threshold (gain) distributions, which again appear lognormally distributed, with smaller widths than spike rate distributions. “

 The author keeps stating that she documents lognormal distributions. But I did not find any statistical test. It should be clear how this has been assessed. Do we know it is not e.g. a gamma distribution?

**Conclusion:**

 “(b) created a generic neural network model to show that these distributions arise from Hebbian learning, and specifically that intrinsic plasticity must be Hebbian as well; “

 This "Hebbian finding" has been repeated many times in the manuscript, and generally there is a problem with the logic. Just because Hebbian learning results in lognormal distributions, it does not imply that neuronal networks must be Hebbian. Other types of learning may also result in lognormal distribution. Can we really make such an exclusive statement based on this model? I suggest a milder version of the manuscript, where it is suggested that Hebbian learning could explain the lognormality seen in real networks.

**Minor issues:**

****

**Abstract:**

 “In this paper, we document lognormal distributions for spike rates,… “  -> In this paper, we investigate distributions for spike rates,… 

 Regarding the statement:

 “Secondly, we created a generic neural model to show that Hebbian learning will create and maintain lognormal distributions.”

 If there is a “secondly…” there should also be a “Firstly,…” Maybe put that before “The difference between….”?

 I also suggest the replacement:  “Secondly, we created a generic neural model to test whether Hebbian learning will create and maintain lognormal distributions.”

 I also suggest replacing:

 “We could prove with the model that not only weights, but also intrinsic gains, need to have strong Hebbian learning in order to produce and maintain the experimentally attested distributions. This settles a long-standing question about the type of plasticity exhibited by intrinsic excitability. 

 “

 with

 “In our model we found that not only weights, but also intrinsic gains, need to have strong Hebbian learning in order to produce and maintain the experimentally attested distributions. This suggest a solution to a long-standing question about the type of plasticity exhibited by intrinsic excitability. “

**Introduction:**

 In the first paragraph I suggest citing

 Wohrer, A., Humphries, M. D., and Machens, C. K. (2013). Population-wide distributions of neural activity during perceptual decision-making. Prog Neurobiol, 103, 156–193.

 because they discuss exactly this issue of distributions and mean of spiking.

 Regarding:

 “With the current data, we show that the distribution of spike rates within any neural tissue follows a power-law distribution, i.e. a dis- tribution with a ‘heavy tail’. There is also a small number of very low-frequency neurons, so that we have a lognormal distribution ^2^. This lognormal distribution is present in spontaneous spike rates as well as under behavioral stimulation. For each neuron, the devia- tion from the mean rate attributable to a stimulus is small (CV = 0.3–1, standard deviation = 1–4 spikes/s), when compared to the variability in mean spike rate over the whole population (5-7-fold), cf. Table 1 and Table 3. “

 First I suggest replacing “any neural tissue” with “many neural tissues”, since no one has looked at all tissue in the nervous system. 

 Further I suggest including the reference: 

 Petersen, P. C. and Berg, R. W. (2016). Lognormal firing rate distribution reveals prominent fluctuation-driven regime in spinal motor networks. eLife, 5, e18805.

 since this will make the statement stronger.

 Another relevant paper which should be discussed and included is:

 Ikegaya Y, Sasaki T, Ishikawa D, Honma N, Tao K, Takahashi N, Minamisawa G, Ujita S, Matsuki N. 2013. Interpyramid spike transmission stabilizes the sparseness of recurrent network activity. Cerebral Cortex 23:293– 304 

 And also the detailed analysis of firing rate versus irregularity for different brain regions and animals should be included: http://www.jneurosci.org/content/36/21/5736

 Regarding this statement:

 “We have constructed a generic model for neuronal populations with adaptable weights and gains. “

 I suggest writing a sentence before that giving a motivation for constructing a model, which seem to come out of the blue as it is. Perhaps something like this: “In order to understand these lognormal distributions we constructed a model….”

**Discussion:**

 Suggest modifying: 

 “It has been suggested that lognormal spike distributions are only a feature of cortical tissue and arise from the strong excitatory recurrent connectivity, but this is neither experimentally nor theoretically correct. “

 ->

 “It has been suggested that lognormal spike distributions are only a feature of cortical tissue and arise from the strong excitatory recurrent connectivity, but this has not been substantiated neither experimentally nor theoretically. “

I have read this submission. I believe that I have an appropriate level of expertise to confirm that it is of an acceptable scientific standard, however I have significant reservations, as outlined above.
